# Outcomes of Percutaneous Stabilization for Pelvic Metastatic Bone Disease: A Systematic Review and Meta-Analysis

**DOI:** 10.3390/cancers18172804

**Published:** 2026-08-28

**Authors:** Alyssa A. Federico, Zachary A. Carter, Alexander W. Iwasyk, Golpira Elmi Assadzadeh, Michael J. Monument, Matthew G. Cable, Joseph K. Kendal

**Affiliations:** 1 Cumming School of Medicine, University of Calgary, Calgary, AB T2N 4N1, Canada; alexander.iwasyk@ucalgary.ca (A.W.I.); mjmonume@ucalgary.ca (M.J.M.); jkkendal@ucalgary.ca (J.K.K.); 2 Department of Surgery, Cumming School of Medicine, University of Calgary, Calgary, AB T2N 2T9, Canada; golpira.elmiassadzad@ucalgary.ca; 3 Arnie Charbonneau Cancer Institute, Cumming School of Medicine, University of Calgary, Calgary, AB T2N 4N1, Canada; 4 McCaig Institute for Bone and Joint Health, Cumming School of Medicine, University of Calgary, Calgary, AB T2N 4Z6, Canada; 5 School of Medicine, Louisiana State University Health Sciences Center, New Orleans, LA 70112, USA; zcart1@lsuhsc.edu (Z.A.C.); mcabl1@lsuhsc.edu (M.G.C.); 6 Department of Orthopaedic Surgery, School of Medicine, Louisiana State University Health Sciences Center, New Orleans, LA 70112, USA

**Keywords:** metastatic bone disease, pelvis, surgery, percutaneous, minimally invasive, outcomes, complications

## Abstract

Background/Objectives: Metastatic bone disease (MBD) of the pelvis is commonly managed with open reconstruction despite substantial morbidity and high complication rates ranging from 16–32%. Percutaneous fixation techniques have emerged as a lower morbidity alternative; however, robust evaluations of outcomes remain limited. Therefore, the primary aim of this systematic review was to evaluate changes in pain and function following percutaneous fixation for pelvic MBD, and to determine the complication and reoperation rates of these procedures. Methods: A systematic review and meta-analysis was conducted according to the Preferred Reporting Items for Systematic Reviews and Meta-Analyses guidelines. MEDLINE, Embase, Scopus, and Cochrane Library databases were searched from inception through 15 May 2025. Studies evaluating percutaneous fixation for pelvic MBD in adults were included. Pooled estimates of visual analog scale (VAS) pain scores, Eastern Cooperative Oncology Group (ECOG) performance status scores, complication rates, and reoperation rates were generated using meta-analysis techniques. Results: Twenty-five studies were included in the final analysis. VAS pain scores were reported in 15 studies, with an average decrease of 5.0 points (95% CI: 3.9–6.1) from pre- to postoperative. ECOG performance status scores were reported in 8 studies, with an average improvement of 1.1 points (95% CI: 0.7–1.5) from pre- to postoperative. Complications were reported in 24 studies, with a pooled complication rate of 8% (95% CI: 6–12%) and reoperation rate of 4% (95% CI: 2–7%). Conclusions: Percutaneous fixation for pelvic MBD provides meaningful improvements in pain and function with relatively low complication rates. However, substantial study heterogeneity exists in the patient population, surgical techniques, and implant selection, reflecting the novelty of this treatment strategy and the absence of standardized guidelines.

## Simple Summary

Metastatic bone disease of the pelvis is an end-stage manifestation of cancer that can cause severe pain, fractures, and loss of mobility. Traditional open surgery can help stabilize the pelvis but is associated with major complications. Emerging technologies have enabled the increased use of minimally invasive percutaneous fixation as an alternative to help lower complication rates, but the outcomes following these procedures have not been comprehensively evaluated. Therefore, the primary aim of this systematic review was to evaluate changes in pain and function following percutaneous fixation for pelvic metastatic bone disease, and to determine the complication and reoperation rates of these procedures. We found that these surgeries were associated with significant improvements in pain and functional status. The complication and reoperation rates were 8% and 4%, respectively. This early data suggests that percutaneous fixation is a safe and effective treatment option for carefully selected patients with pelvic metastatic bone disease.

## 1. Introduction

Metastatic bone disease (MBD) is a major cause of morbidity in patients with advanced cancer, frequently occurring from breast, lung, and prostate cancer [1,2]. MBD substantially impairs quality of life through pain, pathologic fractures, and loss of mobility [3,4,5,6,7]. Management is multidisciplinary and generally palliative, although improving survival rates have increased the importance of selecting surgical treatments that provide durable symptom relief and mechanical stability [6,8,9].

Alongside the axial skeleton, the pelvis is among the most common sites of MBD [10,11]. Pelvic MBD presents as a particular treatment challenge because it can cause severe pain, impaired ambulation, and mechanical instability. Surgical intervention may be indicated for impending or established pathologic fracture and intractable pain [12,13,14]. Destructive pelvic lesions have traditionally been managed with complex open reconstruction, often involving total hip arthroplasty [13,15]. These procedures are associated with relatively high perioperative complication rates, ranging from 16–32%, with dislocation and infection being the most common events [13,16,17,18,19]. When surgical complications arise, systemic treatment may be interrupted [20,21]. Interventional radiology (IR) techniques, such as cementoplasty and ablation, provide a less invasive option for pain relief but may be insufficient in mechanically unstable lesions at risk of fracture [22,23,24].

Percutaneous fixation has emerged as a lesion stabilization option for select patients with MBD [25]. The minimally invasive nature of these procedures may reduce surgical morbidity and enable hip preservation in patients without pre-existing arthritis or disease extension into the articular surface [25,26,27,28]. These techniques commonly use image-guided placement of cannulated screws to bridge fractures or osteolytic defects, providing stability to the pelvic ring and acetabular columns [25,26,29]. Photodynamic nails provide another percutaneous implant option and utilize light-sensitive intramedullary polymer implants that conform to the complex pelvic anatomy [30]. Percutaneous stabilization techniques have been applied across periacetabular and non-periacetabular pelvic lesions and may be combined with interventional procedures [31,32].

Despite increasing use of percutaneous fixation for pelvic MBD lesions, the clinical outcomes and safety of these techniques have not been comprehensively synthesized. A recent narrative review identified 10 studies evaluating percutaneous fixation for periacetabular MBD; however, it did not systematically evaluate clinical outcomes across all pelvic regions or capture all the relevant literature [33]. Additionally, no previous systematic review has comprehensively evaluated changes in patient outcomes and complication rates. Therefore, the primary aim of this systematic review and meta-analysis was to evaluate changes in pain and functional status following percutaneous fixation for pelvic MBD, and to determine the complication and reoperation rates of these procedures. The secondary aim was to determine if these outcomes differed according to implant type.

## 2. Materials and Methods

### 2.1. Search Strategy

The protocol was defined a priori and registered on PROSPERO (registration number: CRD420251079632). Database searches were conducted on 15 May 2025 according to the Preferred Reporting Items for Systematic Reviews and Meta-Analyses (PRISMA) guidelines [34] and the PRISMA 2020 checklist is provided as Supplementary File S1. A librarian at the University of Calgary Health Sciences Centre was consulted for assistance with developing the search strategy. MEDLINE, Embase, Scopus, and Cochrane Library databases were searched with no date or language restrictions. Search terms included metastatic bone disease, pelvis, and percutaneous fixation, as well as multiple synonyms. All final search strategies and numbers of results are reported in Appendix A (Table A1).

### 2.2. Study Eligibility

Studies were included if they met the following criteria: (a) primary research studies, (b) human-based studies, (c) adult population (≥18 years of age), (d) metastatic bone disease population, (e) tumors located in the ilium, ischium, pubis, or acetabulum, and (f) evaluating percutaneous fixation or reconstructive surgical techniques. The exclusion criteria were as follows: (a) non-English papers, (b) non-primary research studies (e.g., conference abstracts, review papers, response letters, editorials), (c) case reports and case series with <10 patients, (d) animal studies, (e) pediatric population, (f) primary bone malignancy population, (g) benign bone tumor population, (h) tumors located in the spine, sacrum, and extremities, and (i) evaluating interventional radiology procedures without lesion fixation (e.g., radiofrequency ablation, cryoablation, cementoplasty, ultrasound therapy, injections, embolization).

### 2.3. Screening

Database search results were uploaded into Covidence (Veritas Health Innovation Ltd., Melbourne, Australia) for review by two independent reviewers (Z.A.C. and A.W.I.) [35]. All titles and abstracts were screened, and those deemed relevant or potentially relevant were included for full text review. Discrepancies were resolved by a third reviewer (A.A.F.). Additionally, the reference lists of papers included in the final analysis and related systematic reviews were screened for additional studies.

### 2.4. Data Extraction

Two independent reviewers (Z.A.C. and A.W.I.) extracted the data for each paper included in the final analysis onto Google Sheets (Alphabet Inc., Mountain View, CA, USA). The extracted data included title, author, year, journal, study design, level of evidence, country, time frame of data collection, population, inclusion and exclusion criteria, follow-up period, sample size, number of males and females, age, type of primary malignancy, tumor location, procedure indication, type of procedure, number of procedures performed, type of implant used, number of implants per procedure, diameter and length of screws used, additional hardware placed, procedure duration, number and type of associated IR procedures, hospital length of stay, number of readmissions, number of admissions to the intensive care unit (ICU), pre- and postoperative visual analog scale (VAS) pain scores, pre- and postoperative Eastern Cooperative Oncology Group (ECOG) performance status scores, number and type of complications, and number and type of reoperations. The type of provider performing the procedures was recorded and categorized as orthopaedic surgeon or IR physician. If not reported in the methods, the specialty of the senior author was used as a proxy for the type of provider. Complications were excluded if they were asymptomatic, reflected disease-related progression without mechanical failure, or unrelated to the procedure. All data extraction was verified by a third, independent reviewer (A.A.F.).

### 2.5. Implant Types

All studies were categorized based on the type of implants used during the procedures. The categories included cement-augmented screws, non-augmented screws, both screw types, and photodynamic nails (IlluminOss Medical, East Providence, RI, USA). For the purposes of this review, screws were considered cement-augmented if cement was injected through the screw cannula during insertion or was aimed directly at the screw tip. For studies categorized as using both screw types, cement augmentation was selectively performed when clinically indicated, such as in patients with poor screw purchase from osteopenia or osteolysis. Therefore, these cohorts contained both patients treated with cement-augmented and non-augmented screws [28,31,36,37,38].

### 2.6. Patient Outcomes

Patient outcomes were assessed using the VAS pain scores and ECOG performance status because these were the most consistently reported and clinically relevant outcome measures across the included studies. The VAS pain score is measured on a scale of either 0 to 10 or 0 to 100, with higher scores indicating more severe pain [39]. When reported on a 0 to 100 scale, VAS scores were converted to the equivalent 0 to 10 scale for analysis. The minimal clinically important difference (MCID) for VAS pain scores when assessing postoperative pain reduction in ranges from 1 to 2.3 points [40,41,42]. The ECOG performance status score assesses functional capacity on a scale from 0 to 5, with 0 representing full performance of pre-disease activities and 5 representing death. In other words, a lower score indicates better functional status [43]. The MCID for ECOG performance status is a change of 1 point [44]. For all outcomes, the values represent the score obtained at the final postoperative follow-up reported in each paper.

### 2.7. Quality Assessment

Quality assessment was performed by two independent reviewers (Z.A.C. and A.W.I.). The Methodological Index for Non-Randomized Studies (MINORS) criteria were used to assess non-randomized studies [45,46]. Non-comparative studies were graded out of 16 with a score of ≤8 indicating poor quality, 9–14 indicating moderate quality, and 15–16 good quality [45,46]. The Interclass Correlation Coefficient (ICC) was used to calculate interrater agreement for the MINORS quality assessment. Standardized score classification was followed whereby <0.50 indicated poor agreement, 0.50 to 0.75 indicated moderate agreement, 0.75 to 0.90 indicated good agreement, and ≥0.90 indicated excellent agreement [45,47].

### 2.8. Statistical Analysis

Descriptive analyses were performed using Python (version 3.9.10). Continuous data were reported as medians with interquartile ranges or ranges and were converted to means and standard deviations using established formulas [48]:

Mean = (Median + Q1 + Q3)/3

and

SD = (Q3 − Q1)/1.35 or SD = (Max − Min)/4.

Meta-analyses were conducted in R (version 4.4.1). Continuous outcomes were analyzed as means, pre-post outcomes as mean differences, and dichotomous outcomes as proportions. For continuous and pre-post outcomes, random-effects models were used with between-study variance estimated via restricted maximum likelihood. Dichotomous outcomes were analyzed using Generalized Linear Mixed Models with random study effects modeled directly. Heterogeneity was quantified using the inconsistency index (I^2^) and assessed using the Chi-squared test, with I^2^ > 50% indicating substantial heterogeneity. Effect estimates are reported as pooled means, mean differences, proportions, or relative risks with 95% confidence intervals (CIs). Statistical significance was defined as *p* < 0.05. Forest plots were generated for all outcomes, and subgroup analyses were conducted based on implant type.

Sensitivity analysis was conducted using a leave-one-out approach, in which each study was sequentially excluded to assess its influence on the pooled estimates and between-study heterogeneity. Univariable random-effects meta-regression analyses were performed using restricted maximum likelihood to assess whether study-level characteristics explained between-study heterogeneity in the estimated changes in VAS pain and ECOG performance status scores. The mean difference in scores (pre- to postoperative) was used as the effect estimate with the corresponding standard error. The variables examined were implant type, age, follow-up duration, tumor location, and number of IR procedures. For categorical variables, coefficients were interpreted relative to the reference category. For each model, the overall test of the variable was used to assess its association with the observed effect sizes. The proportion of between-study heterogeneity explained by each variable was summarized using R^2^ values, and residual heterogeneity was assessed using I^2^ values. Given the limited number of available studies and incomplete reporting of several study-level characteristics, multivariable meta-regression was not performed to avoid model overfitting and unstable parameter estimates.

## 3. Results

### 3.1. Search Results

**Figure 1:**
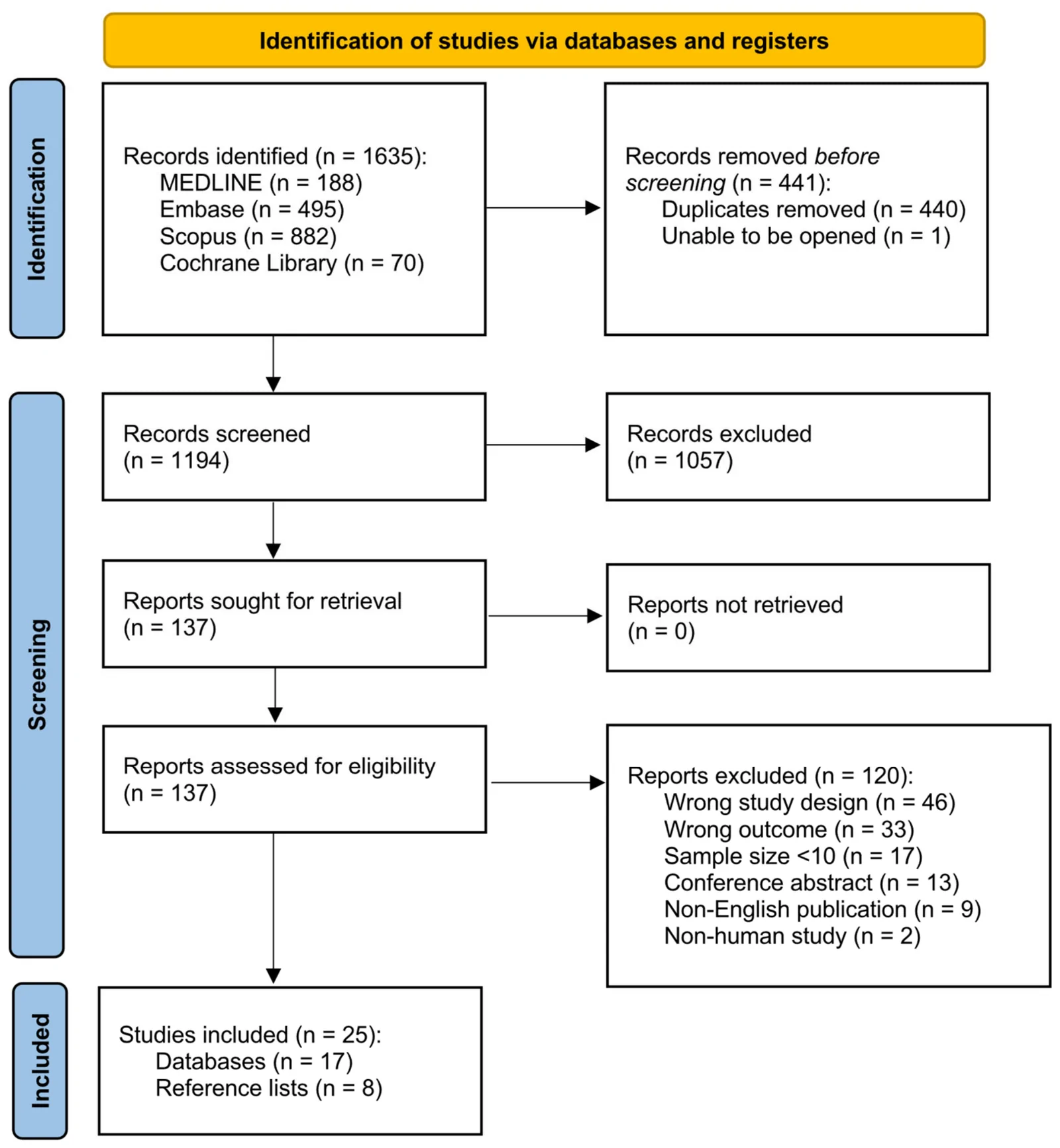
PRISMA flowchart of study database search and review process.

There were 1635 studies identified by the database searches (Figure 1). There were 440 duplicates identified and removed, and the remaining abstracts were screened. During abstract screening, 152 conflicts were resolved by a third reviewer, and 137 studies were identified as potentially relevant and underwent full-text review. Of these studies, 17 met the final inclusion criteria. Additionally, eight papers that met the inclusion criteria were identified through reference list screening and review of other related systematic reviews. Overall, there were 25 studies included in the final analysis [25,26,27,28,29,30,31,32,36,37,38,49,50,51,52,53,54,55,56,57,58,59,60,61,62].

**Figure 2:**
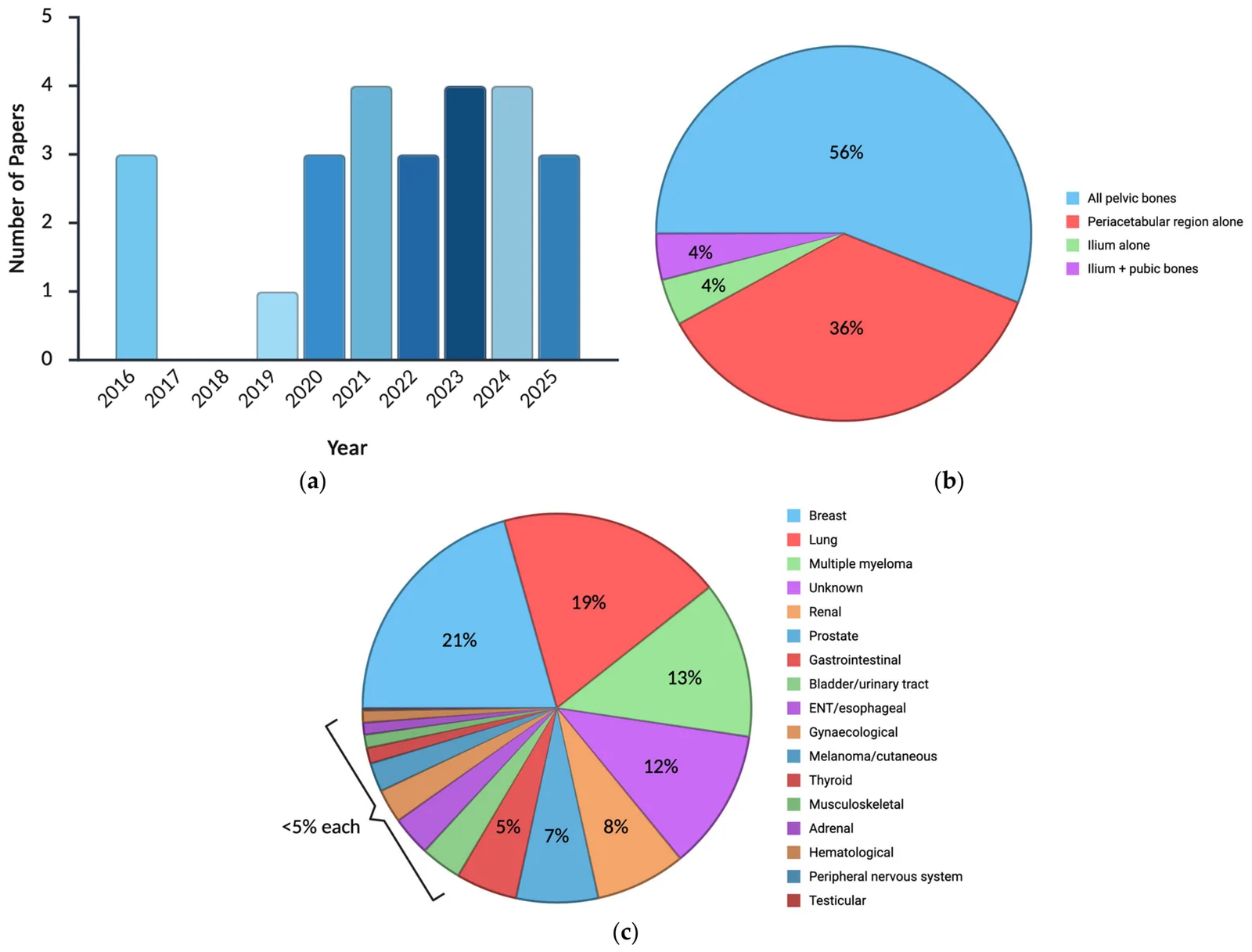
Summary of study and patient characteristics. Distribution of publications by year (**a**), proportion of lesion locations included in studies (**b**), and proportion of patients with each type of primary malignancy (**c**).

All studies were conducted in the past 10 years, from 2016 to 2025 (Figure 2a). There were 14 studies (56%) completed in the United States, 9 studies (36%) in France, and 2 studies (8%) in China. In terms of study design, there were 18 retrospective cohort studies (72%), four prospective cohort studies (16%), two retrospective case series (8%), and one prospective case series (4%) (Table 1).

### 3.2. Patient Characteristics

A total of 1034 patients were included, with a mean age of 64.5 ± 3.6 years and a follow-up period of 9.7 ± 6.2 months. The sex distribution was nearly equal, with 52.6% female and 47.5% male participants. Most studies evaluated lesions in any region of the pelvis (56%), while a smaller proportion focused solely on the periacetabular region (36%), ilium (4%), or ilium and pubis (4%) (Figure 2b). There were 12 studies that included lesions in locations other than the pelvis such as the sacrum, femur, scapula, spine, sternum, clavicle, mandible, shoulder, tibia, and calcaneus. The most common primary malignancies were breast (20.6%), lung (18.7%), multiple myeloma (13%), unknown/unspecified (11.7%), prostate (6.8%), and renal (7.5%). All other types of primary malignancy occurred in less than 5% of patients (Figure 2c).

### 3.3. Procedure Characteristics

The procedure indications were impending fracture in 16% of studies, pathologic fracture in 8% of studies, and impending or pathologic fracture in 76% of studies. The mean procedure duration was 132.9 ± 42.2 min with 2.2 ± 0.6 implants placed per procedure. Four studies were focused on photodynamic nail fixation (16%), while the rest used screw fixation techniques (84%). The mean length of hospital stay amongst all studies was 3.5 ± 2.6 days. Among the included studies, 15 (60%) had an orthopaedic surgeon and 10 (40%) had an IR physician as the performing provider or senior author. Nine of 15 orthopaedic-led studies (60%) were published from 2023 onward, compared with 2 of 10 IR-led studies (20%).

Of the studies that focused on screw fixation, 48% used cement-augmented screws, 16% used non-augmented screws, and 20% used a combination of cement- and non-augmented screws. Screw diameters ranged from 4 mm to 8 mm, and the mean length of screws used was 102 ± 12.2 mm. A small proportion of patients (4.2%) had additional implants placed under the same anaesthetic for additional lesions, including hip arthroplasty, femoral intramedullary nail, and femoral percutaneous screws. More than half of patients (57.7%) underwent adjunct interventional procedures, including lesion cementoplasty, balloon osteoplasty, ablation, and embolization.

### 3.4. Pain

**Figure 3:**
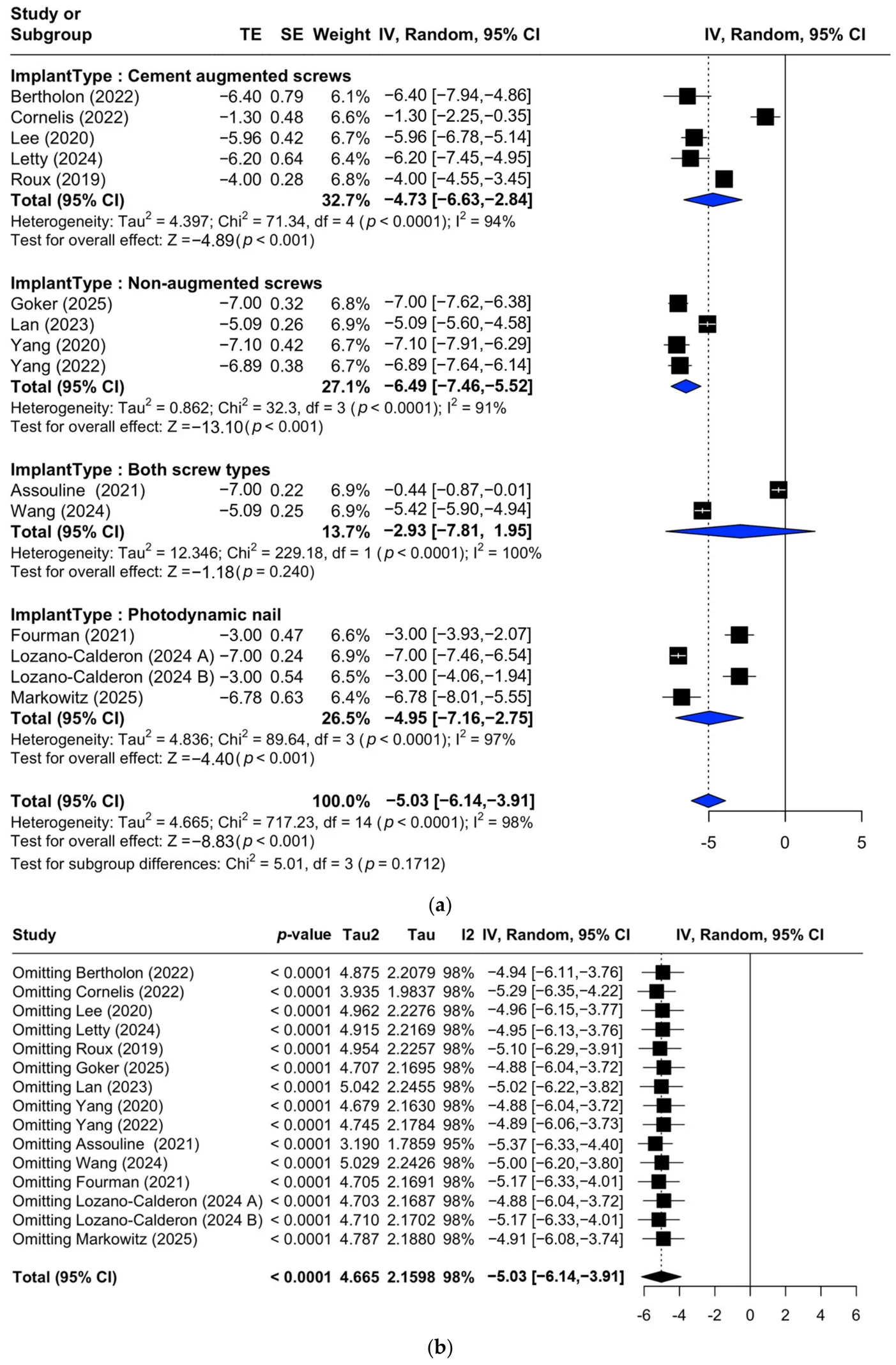
Forest plots showing the change in VAS pain scores from preoperative to postoperative assessment: (**a**) subgroup analysis by implant type and (**b**) leave-one-out sensitivity analysis [25,27,30,32,36,38,51,52,55,56,58,59,60,61,62].

The outcomes reported in each study are summarized in Table 2. There were 15 studies (60%) that reported pre- and postoperative VAS pain scores, with a collection range of 4 weeks to 12 months postoperative and total sample size of 499 patients. Five papers did not specify the postoperative timepoint when scores were collected. The mean VAS pain score decreased by 5.0 points (95% CI: 3.9–6.1) when comparing pre- to postoperative measures (Figure 3a). Despite substantial heterogeneity (I^2^ = 98%), the magnitude of improvement exceeded the MCID of 1 to 2.3 points across studies [40,41,42,63,64].

The leave-one-out sensitivity analysis demonstrated that the pooled effect estimate remained consistent following sequential exclusion of each individual study, with estimates ranging from 4.9 to 5.4 and all 95% CIs excluding zero (Figure 3b). This suggests that no single study substantially influenced the direction or statistical significance of the pooled effect.

Univariable meta-regression analyses were performed to explore potential sources of heterogeneity. Implant type (*p* = 0.25), treatment group (*p* = 0.75), age (*p* = 0.30), follow-up duration (*p* = 0.58), and tumor location (*p* = 0.86) were not significantly associated with the pooled change in VAS pain scores. The number of adjunctive IR procedures was the only significant moderator of change in VAS pain scores (β = 0.11, 95% CI: 0.025–0.20; *p* = 0.012), accounting for 32.8% of the estimated between-study heterogeneity. Studies incorporating a greater number of adjunctive IR procedures demonstrated slightly smaller improvements in VAS pain scores; however, the magnitude of this association was small and substantial residual heterogeneity remained (I^2^ = 96.1%).

### 3.5. Functional Status

**Figure 4:**
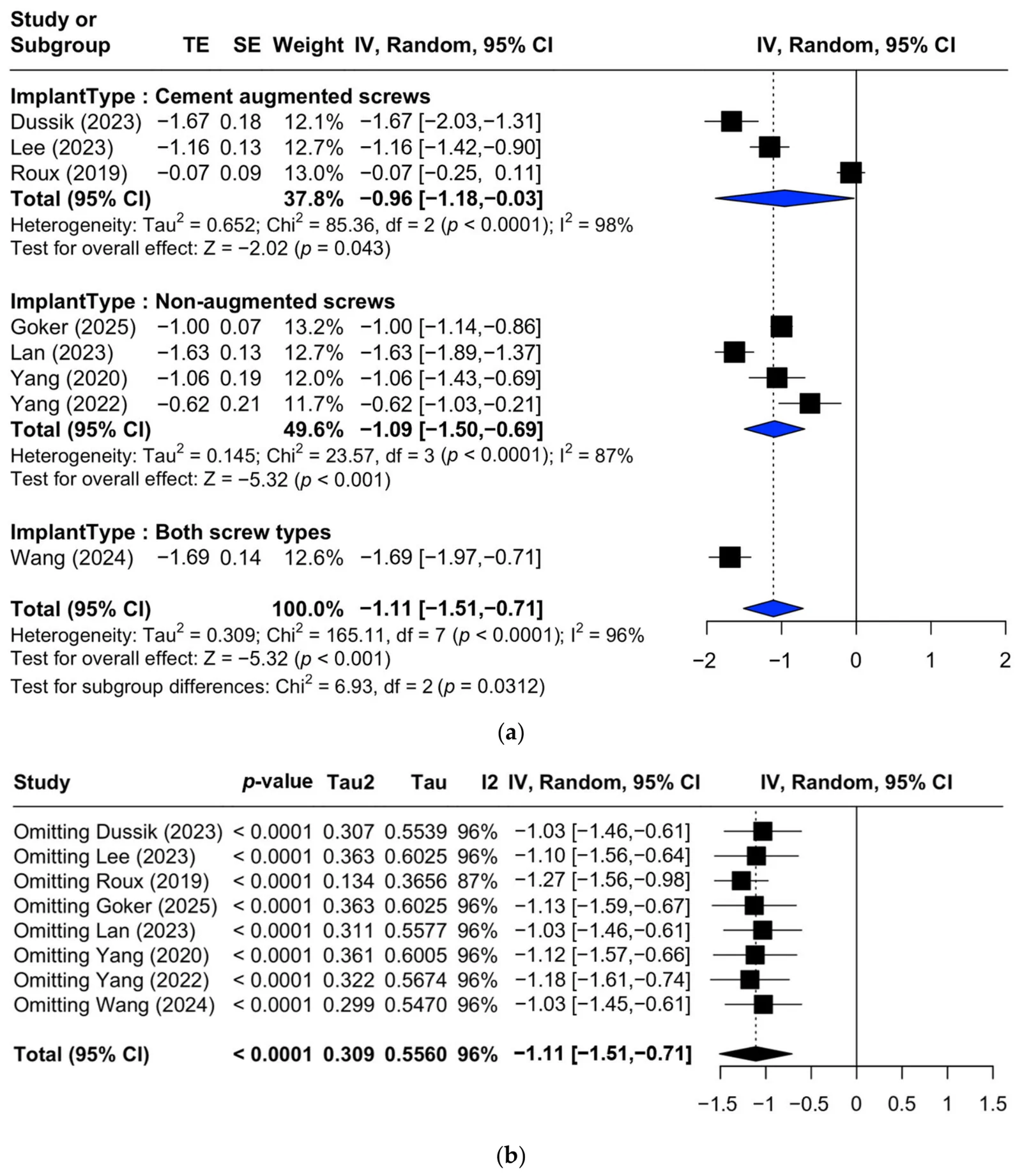
Forest plots showing the change in ECOG performance status scores from preoperative to postoperative assessment: (**a**) subgroup analysis by implant type and (**b**) leave-one-out sensitivity analysis [27,38,50,52,55,57,61,62].

There were 8 studies (32%) that reported pre- and postoperative ECOG performance status scores with a total of 374 patients. Notably, no studies using photodynamic nails reported ECOG scores. The collection time points ranged from 2 weeks to 12 months postoperative, with three papers not specifying the time. The mean ECOG performance status scores improved by 1.1 points (95% CI: 0.7–1.5) when comparing pre- to postoperative measures (Figure 4a). This change is above the MCID of 1 point, indicating a meaningful improvement in patient quality of life [44]. The I^2^ statistic for this analysis was 96%, indicating a high degree of heterogeneity [63,64].

The leave-one-out sensitivity analysis demonstrated that the pooled effect estimate remained consistent following the sequential exclusion of each individual study, with estimates ranging from 1.0 to 1.3 and significant 95% CIs (Figure 4b). Once again, this analysis demonstrated that no single study substantially influenced the pooled effect and that the results of this analysis were robust.

The univariable meta-regression analyses found no statistically significant evidence that implant type (*p* = 0.57), age (*p* = 0.93), follow-up duration (*p* = 0.61), tumor location (*p* = 0.22), or associated IR procedures (*p* = 0.30) explained the observed between-study heterogeneity. Across all models, there remained substantial heterogeneity (I^2^: 86.6–96.0%).

### 3.6. Complication Rate

**Figure 5:**
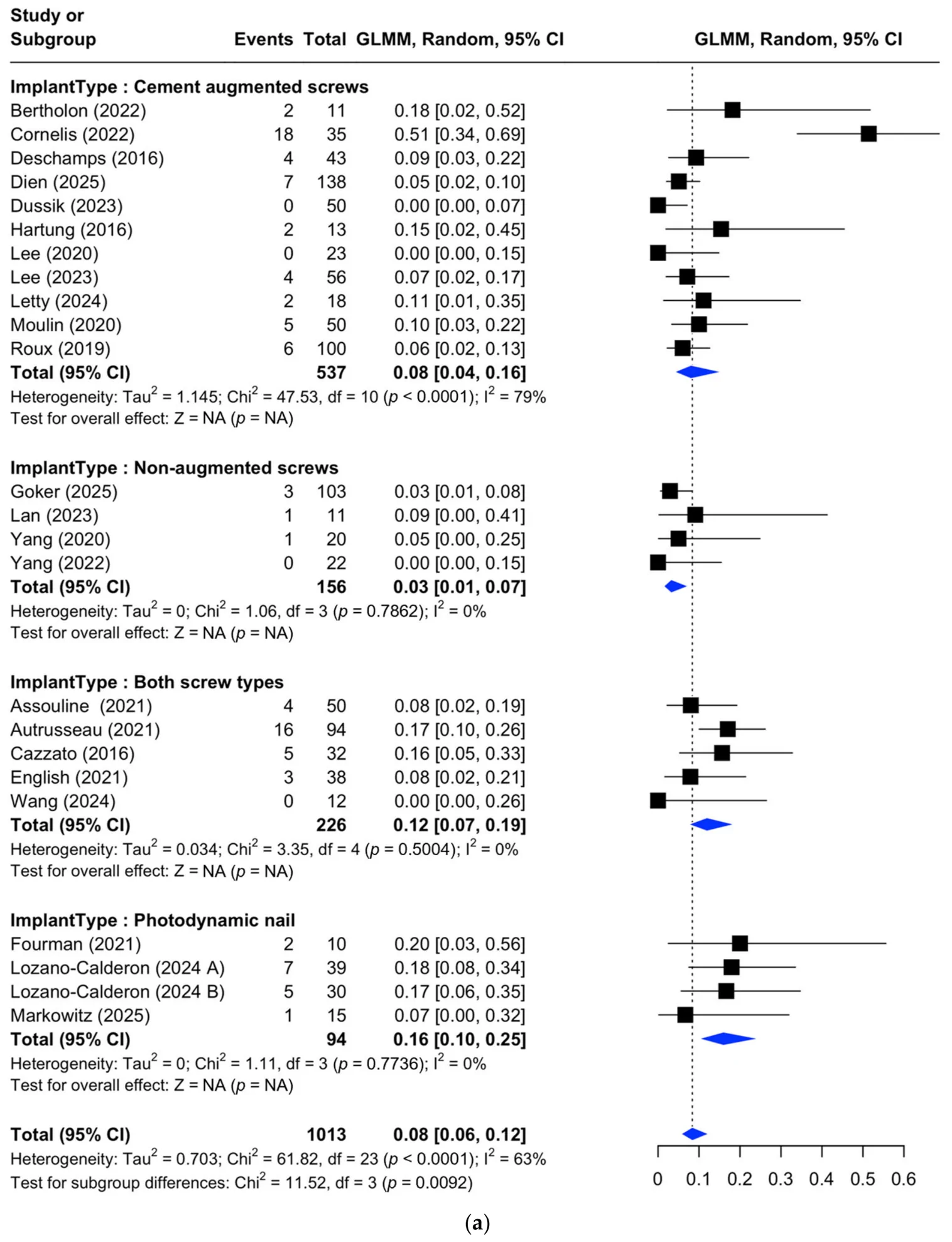

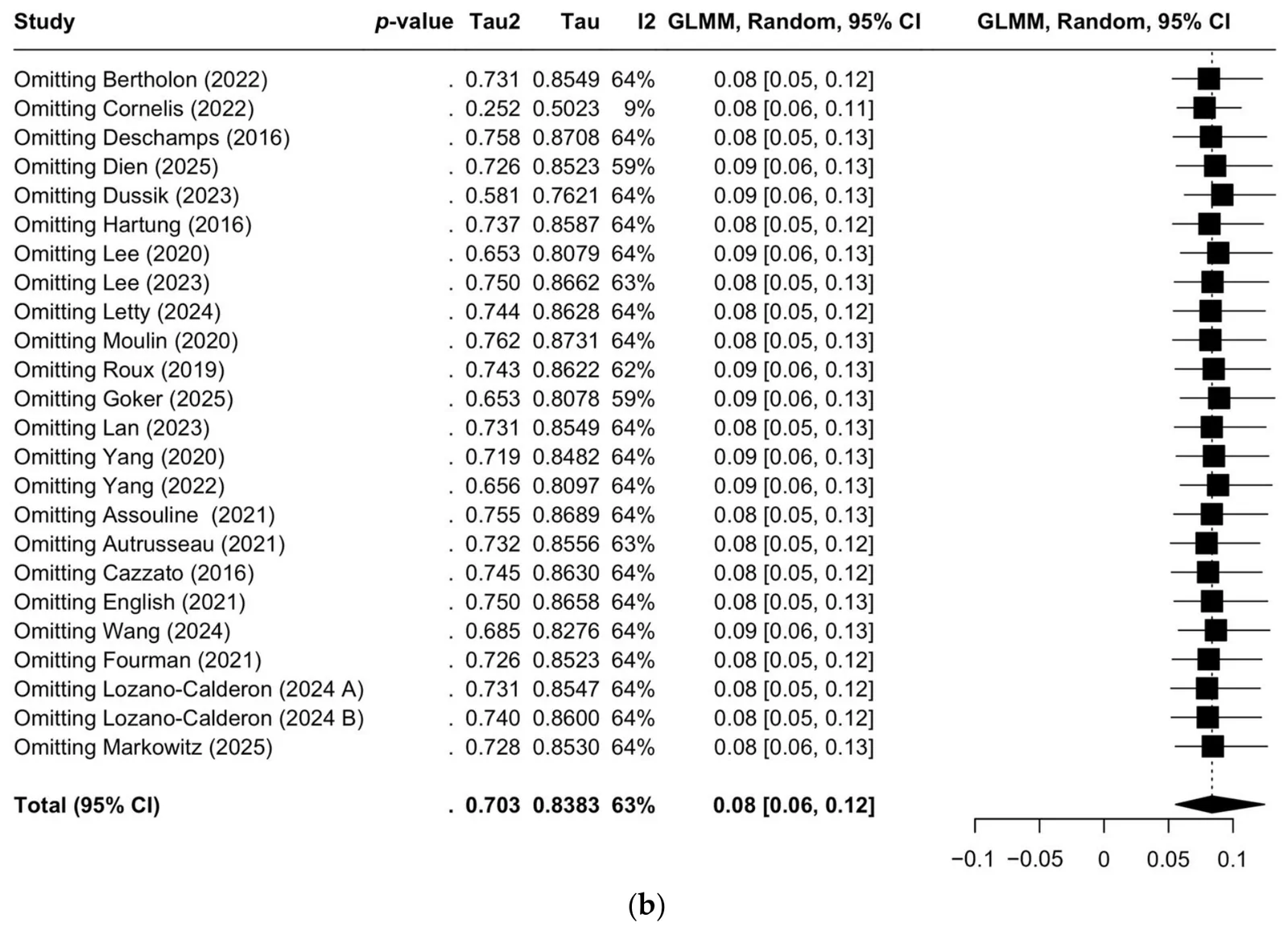
Forest plots of complication rates: (**a**) subgroup analysis by implant type and (**b**) leave-one-out sensitivity analysis [25,26,27,28,29,30,31,32,36,37,38,49,50,51,52,53,55,56,57,58,59,60,61,62].

Complications were reported in 24 studies (96%) and the pooled complication rate was 8% (95% CI: 6–12%) (Figure 5a). The three most common complications included hematoma (3.5%), implant loosening (1.1%), and infection (1.0%). All other complications occurred in less than 1% of patients and included deep vein thrombosis, nerve injury, wound dehiscence, hardware pain, pulmonary embolism, hip septic arthritis, broken hardware, arterial injury, unspecified technical failure, intra-articular hardware, intraoperative fracture, non-union, and arterial thrombosis. The I^2^ statistic for this analysis was 62.8%, indicating a moderate to high degree of heterogeneity in the data [63,64].

The leave-one-out sensitivity analysis showed minimal variation when removing each individual study from the analysis, with complication rates ranging from 8–9% with significant 95% CIs (Figure 5b). Although Cornelis et al. (2022) was a major contributor to between-study heterogeneity in complications, exclusion of this study did not alter the pooled estimate, indicating that the overall findings were robust [25].

### 3.7. Reoperation Rate

**Figure 6:**
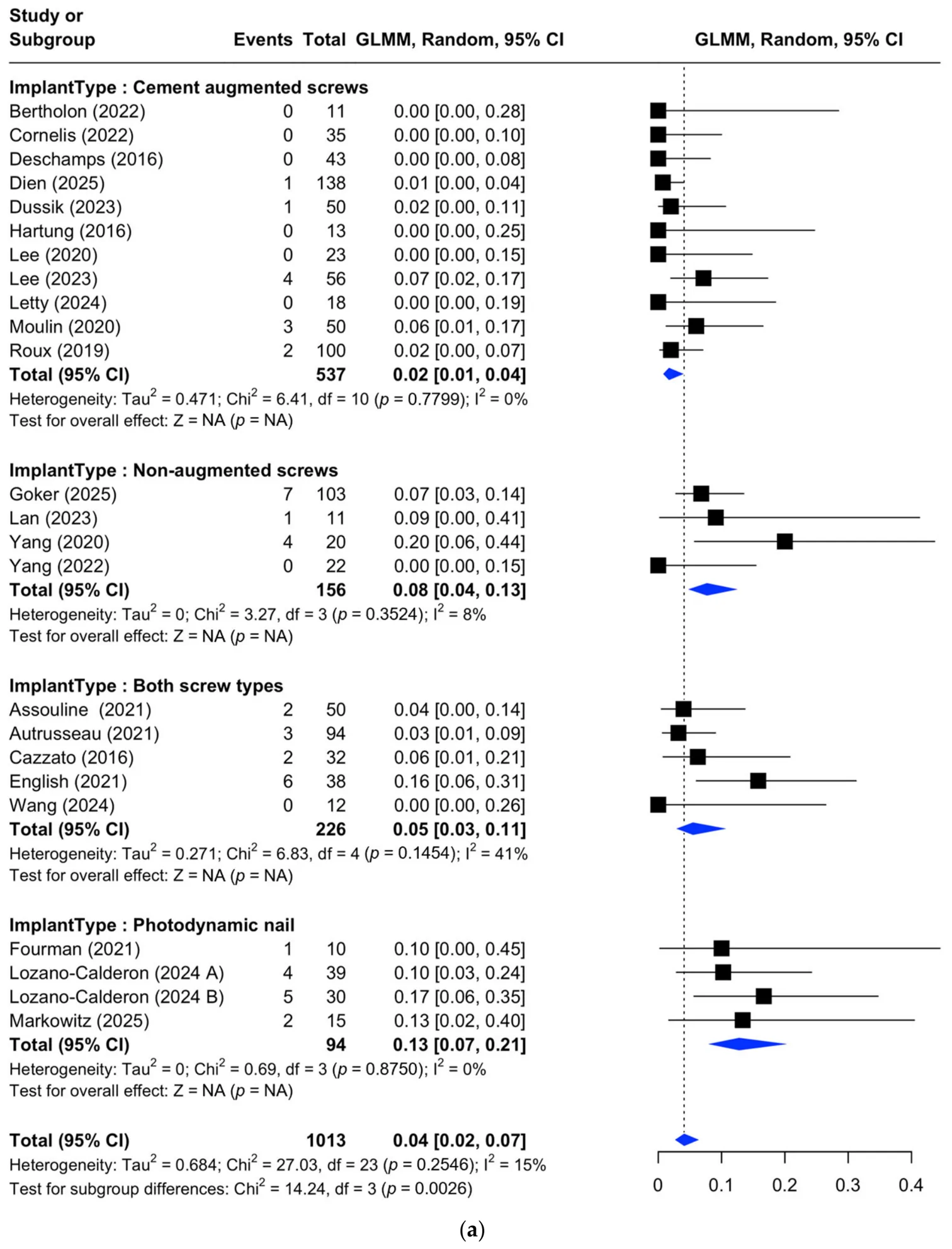

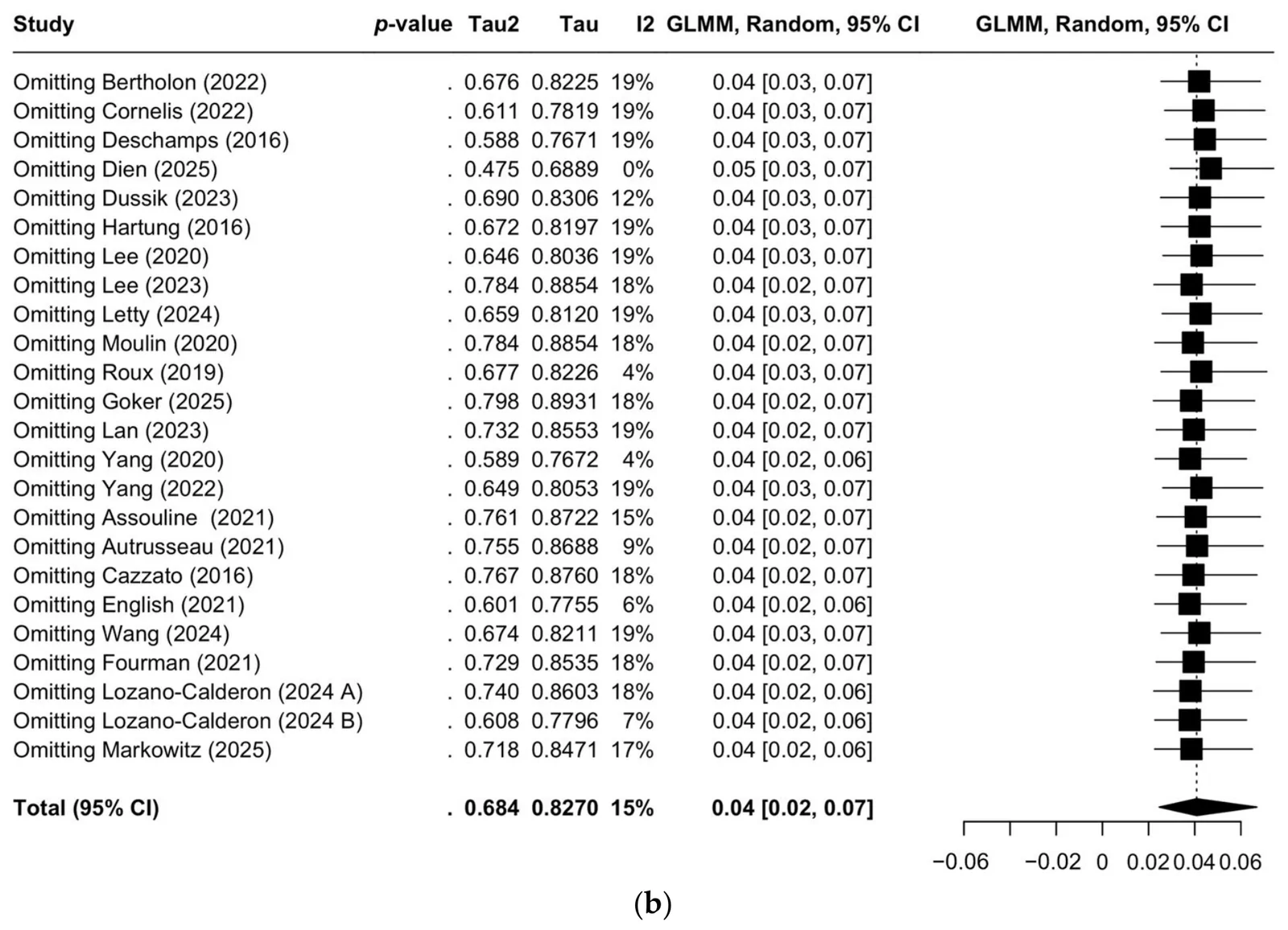
Forest plots of reoperation rates: (**a**) subgroup analysis by implant type and (**b**) leave-one-out sensitivity analysis [25,26,27,28,29,30,31,32,36,37,38,49,50,51,52,53,55,56,57,58,59,60,61,62].

The pooled reoperation rate was reported in 24 studies (96%) and the pooled reoperation rate was 4% (95% CI: 2–7%). Reasons for reoperation included total hip arthroplasty (1.7%), repeat minimally invasive procedures for lesion progression (1.0%), hardware removal (0.8%), wound/infection debridement (0.7%), arterial embolization (0.2%), girdlestone (0.2%), arthrodesis (0.1%), and hemiarthroplasty (0.1%) (Figure 6a). There was a low to moderate amount of heterogeneity in the data, with an I^2^ statistic of 41.9% [63,64].

The leave-one-out sensitivity analysis demonstrated that the pooled effect estimate remained consistent following sequential exclusion of each individual study, with estimates ranging from 4–5% and all 95% CIs excluding zero (Figure 6b). This suggests that no single study substantially influenced the direction or statistical significance of the pooled effect. The degree of heterogeneity decreased when excluding 6 studies; however, the pooled estimates remained the same or within one point of the original value [27,28,31,49,59,61].

### 3.8. Outcomes by Implant Type

Subgroup analyses were performed to assess the differences in primary outcome measures based on implant type. For VAS pain scores, the smallest decrease of 2.9 points (95% CI: 2.0–7.8) was observed in patients who received both cement- and non-augmented screws, and the largest decrease of 6.5 points (95% CI: 5.5–7.5) occurred in patients who received non-augmented screws alone (Figure 3a). There was minimal variation in the change in ECOG scores in studies using different implant types (Figure 4a).

Procedures using photodynamic nails had the highest reported complication rate at 16% (95% CI: 10–25%), including venous thromboembolism (*n* = 7), wound dehiscence (*n* = 3), infection (*n* = 2), implant loosening (*n* = 2), and hardware pain (*n* = 1) (Figure 5a). Procedures using non-augmented screws had the lowest reported complication rate at 3% (95% CI: 1–7%).

The reported rate of reoperation was highest with photodynamic nails at 13% (95% CI: 7–21%), and lowest with cement-augmented screws at 1% (95% CI: 0–5%) (Figure 6a). The reasons for reoperation reported in the photodynamic nail studies included wound or infection debridement (*n* = 5), total hip arthroplasty (*n* = 4), repeat fixation (*n* = 2), and hardware removal (*n* = 1).

### 3.9. Quality Assessment

All studies were non-randomized, non-comparative studies, and assessed using the 16-point MINORS criteria (Appendix B, Table A2). The average MINORS score was 10.3 (range: 7.5–13), indicating moderate quality evidence [45,46]. The ICC for interrater agreement was 0.89, indicating good agreement [45,47].

## 4. Discussion

### 4.1. Pain and Functional Status

In MBD, surgery aims to improve patient quality of life, particularly by alleviating pain and restoring function. Because bone metastases are a hallmark of advanced disease, surgery is generally not performed with curative intent [65]. Therefore, treatment strategies that can achieve these goals while minimizing morbidity are particularly valuable. Percutaneous fixation techniques are an emerging approach to treating MBD lesions in the pelvis that may meet these criteria. Percutaneous fixation has the potential to benefit patients without pre-existing arthritis or extensive articular involvement requiring reconstruction, while preserving the native hip.

When clinically indicated, percutaneous fixation for pelvic MBD may significantly improve pain and functional status, as demonstrated by this meta-analysis. VAS pain scores decreased by 5.0 points (95% CI: 3.9–6.1) and ECOG scores improved by 1.1 points (95% CI: 0.7–1.5) from pre- to postoperative. Both these changes were above the literature reported MCIDs, indicating clinically meaningful differences [40,41,42,44]. However, the very high heterogeneity for VAS (I^2^ = 98%) and ECOG (I^2^ = 96%) indicates the magnitude of improvement varied substantially across studies. Clinically, the pooled estimates should therefore be interpreted as average effects across diverse study populations rather than uniform treatment effects. Despite this variability, the overall direction of effect favored improvements in pain and function, and the sensitivity analysis demonstrated that pooled estimates were robust and not influenced by the inclusion or exclusion of individual studies.

The exploratory meta-regression identified an association between a greater number of adjunctive IR procedures and slightly smaller improvements in VAS pain scores. This finding should not be interpreted as evidence that adjunctive IR procedures reduce the analgesic benefit of fixation. The analysis was performed at the study rather than patient level, and the observed effect was small relative to the overall improvement in pain. Furthermore, the use of cementoplasty, ablation, embolization, or combinations of these techniques may reflect differences in lesion characteristics, disease burden, or procedural complexity, introducing substantial confounding. The magnitude of this association was also small relative to both the overall 5-point improvement in VAS pain and reported MCID thresholds. Considerable residual heterogeneity remained after accounting for this variable, and no corresponding association was observed for ECOG performance status. Accordingly, this finding should be considered hypothesis-generating rather than evidence of a causal relationship between adjunctive IR procedures and postoperative pain outcomes.

### 4.2. Complication Rate

In addition to improving patient outcomes, percutaneous fixation was associated with a pooled complication rate of 8%. Historically, complication rates of 16–32% have been reported following open reconstruction for pelvic MBD [13,16,17,18,19]. However, these estimates should not be directly compared because open reconstruction and percutaneous fixation are generally applied to different patterns and severities of disease.

Recent comparative data provide further context. Dowd et al. (2026) compared open reconstruction with minimally invasive screw fixation with cementation and ablation for periacetabular MBD [66]. Minimally invasive treatment was associated with lower blood loss, transfusion requirements, operative time, and hospital length of stay, while short-term pain and ambulatory outcomes, complications, secondary procedures, and 90-day mortality were similar. Patients undergoing minimally invasive treatment also returned to adjuvant oncologic therapy sooner [66]. Although these findings support a potential morbidity advantage of minimally invasive approaches in appropriately selected patients, treatment selection remains highly dependent on the extent of osseous destruction and articular involvement.

Furthermore, open reconstruction is not the only alternative to percutaneous fixation. Patients with painful pelvic MBD without clinically significant mechanical instability may be appropriately managed with radiotherapy, systemic oncology treatment, or image-guided ablative and cementoplasty procedures [22,23,24]. Conversely, fixation provides an important structural role when pain is associated with mechanical instability. Future studies should focus on defining the patient and lesion characteristics that determine when mechanical stabilization provides additional benefit within a multidisciplinary treatment strategy.

### 4.3. Reoperation Rate

The pooled reoperation rate was 4%, with total hip arthroplasty (1.7%) being the most common subsequent procedure. Indications for arthroplasty included disease progression, femoral head collapse, arthritis, revision of arthroplasty implants placed during index procedure, and fracture. Notably, none of the arthroplasty procedures were performed due to implant failure, although increased risk of disease progression requiring subsequent surgery remains a potential limitation of percutaneous fixation procedures [30,31,52,57,59,60,61]. An additional 1% of patients required repeat percutaneous procedures for disease progression, pathologic fracture through the lesion, hardware pain, and broken or displaced implants. Although some of the reasons for reoperation were the direct result of implant-related issues, these events were uncommon [27,31,36,49,52,57,59]. Importantly, reoperation for wound dehiscence or infection occurred in only 0.6% of patients. This low rate may reflect the limited soft-tissue disruption, short operative times, and low implant burden associated with a minimally invasive approach, although further research is needed to confirm the association in this context.

### 4.4. Implant Selection

The present study shows that there are three main construct types used for percutaneous fixation of pelvic MBD lesions: cement-augmented screws, non-augmented screws, and photodynamic nails. Additionally, a group of papers used either cement-augmented or non-augmented screws depending on the patient, bone quality and lesion type [28,31,36,37,38]. Most studies utilized cement-augmented screws, providing the most data included in the meta-analyses of patient outcomes and complications [23,25,27,29,32,49,50,53,54,56,57,58].

Photodynamic nails had the highest reported complication and reoperation rates; however, interpretation is limited by small sample sizes and study heterogeneity. Wound dehiscence and infection were the most common complications and the leading indications for reoperation. Although the underlying reasons remain unclear, the higher complication rates observed with photodynamic nails may be related to the greater technical complexity and longer operative times associated with their use compared with screw fixation. Additionally, there were higher rates of venous thromboembolism reported in studies using photodynamic nails, which may be a result of different institutional screening practices [30,51,59,60].

The literature also demonstrates an evolving multidisciplinary adoption of percutaneous fixation techniques. Although earlier studies were more commonly led by interventional radiology groups, orthopaedic surgeon-led publications have become increasingly prominent in recent years. This trend may reflect increasing recognition of percutaneous fixation as a reconstructive strategy requiring collaboration and integration of mechanical stabilization with image-guided therapies.

### 4.5. Strengths and Limitations

Strengths of this study include the comprehensive literature search and rigorous manual data extraction process, supporting the completeness and accuracy of the resultant dataset. The sensitivity analysis and meta-regression provide evidence that the pooled estimates for the primary outcomes are robust and not substantially influenced by between-study heterogeneity. As the first systematic review on the subject, this study also addresses an important literature gap and provides the first comparative overview of the available percutaneous fixation techniques for pelvic MBD lesions.

There are important limitations to consider when interpreting these findings. Notable heterogeneity was present across the meta-analyses, as indicated by the moderate to very high I^2^ values, limiting the precision and generalizability of the pooled estimates. Leave-one-out sensitivity analyses demonstrated minimal differences in the pooled estimates when individual studies were removed, indicating that the observed heterogeneity was not driven by any single study. Rather, it likely reflects both the clinical diversity of the MBD population and variation in how percutaneous fixation is performed and evaluated. The MBD population is heterogeneous at both the patient and disease levels, with differences in demographic characteristics, primary malignancy, overall disease burden, lesion location and extent, baseline pain and functional impairment, and prior local or systemic treatment. These factors may influence both patient selection and response to treatment. Percutaneous fixation for pelvic MBD is also relatively novel, and studies varied in patient-selection criteria, procedural techniques, surgical adjuncts, and postoperative assessment timing. The available data did not permit meaningful subgroup analyses, and therefore the relative contributions of these patient-, disease-, and treatment-related factors could not be determined. Although this heterogeneity remains a limitation of the available evidence, it also reflects the clinical diversity of the MBD population and the relative novelty of percutaneous fixation, highlighting the need for standardized patient-selection criteria, procedural reporting, outcome assessment, and treatment guidelines.

Outcome measures were inconsistently reported across studies, limiting both the number of studies that could be included in each analysis and the statistical power of these analyses. Postoperative assessment timing also varied across studies and was not specified in several studies. When multiple postoperative time points were reported, we used the final postoperative score provided in each paper for consistency. Consequently, the pooled analyses may combine outcomes obtained at different stages of postoperative recovery. Differences in outcome measures, reporting practices, and follow-up time points limited the ability to synthesize findings under standardized conditions and could not be fully addressed in this study.

Lastly, the overall methodological quality of the studies was rated as moderate according to the MINORS criteria. All studies were non-comparative, and the majority were retrospective in nature. Given that the original studies did not represent the highest possible level of evidence, the pooled estimates should be interpreted with caution. In addition, there were important confounders that could not be accounted for due to the nature of the data. Twelve studies included some patients with lesions outside the pelvis whose outcomes were combined with those of patients with pelvic lesions and could not be analyzed separately.

## 5. Conclusions

In this study, percutaneous fixation for pelvic metastatic bone disease was associated with meaningful improvements in pain and functional status, with mean improvements exceeding established MCIDs. The pooled complication and reoperation rates were 8% and 4%, respectively. These findings support percutaneous fixation as a joint-preserving, minimally invasive treatment strategy for select patients with pelvic MBD. However, substantial heterogeneity exists in the patient population, surgical techniques, and implant selection, reflecting the novelty of this treatment strategy. As the use of percutaneous fixation continues to increase, future work should focus on standardized patient selection criteria and defining its role within broader multidisciplinary treatment, including radiotherapy, interventional procedures and open reconstruction.

## Figures and Tables

**Table 1:** Summary of study characteristics.

First Author (Year)	Level of Evidence	Study Design	Country	Sample Size	Follow-Up Period (Mean)	Tumor Location	Other Disease Sites (Number of Participants)	Procedure Indication	Implant Type
Assouline (2021) [36]	3	Retrospective cohort	France	50	22 mo.	All pelvic bones	Sacrum (11)	Impending fracture	Both screw types
Autrusseau (2021) [28]	3	Retrospective cohort	France	94	11.8 mo.	All pelvic bones	Sacrum (38) Proximal femur (7) Scapula (5) Spine (4) Sternum (1) Clavicle (1) Mandible (1)	Impending, pathologic, or insufficiency fracture	Both screw types
Bertholon (2022) [32]	2	Prospective cohort	France	11	–	Ilium, pubis	Proximal femur (1) Odontoid (1)	Pathologic fracture	Cement augmented screws
Cazzato (2016) [37]	3	Retrospective cohort	France	32	8.7 mo.	All pelvic bones	Sacrum (5) Femoral neck (6) Shoulder (4) Vertebrae (1)	Impending, pathologic, or insufficiency fracture	Both screw types
Cornelis (2022) [25]	3	Retrospective cohort	United States	35	6 mo. (median)	All pelvic bones		Impending fracture	Cement augmented screws
Deschamps (2016) [26]	3	Retrospective cohort	France	43	2.5 mo.	Ilium	Proximal femur (35)	Impending fracture	Cement augmented screws
Dien (2025) [49]	3	Retrospective cohort	France	138	4.5 mo. (median)	All pelvic bones	Sacral (34) Femoral neck (12)	Impending or pathologic fracture	Cement augmented screws
Dussik (2023) [50]	3	Retrospective cohort	United States	50	11 mo. (median)	Periacetabular region		Impending or pathologic fracture	Cement augmented screws
English (2021) [31]	3	Retrospective cohort	United States	38	9 mo.	Periacetabular Region		Impending or pathologic fracture	Both screw types
Fourman (2021) [51]	4	Retrospective case series	United States	10	–	Periacetabular region	Sacrum (2)	Impending or pathologic fracture	Photodynamic nail
Goker (2025) [52]	3	Retrospective cohort	United States	103	14 mo. (median)	All pelvic bones		Impending or pathologic fracture	Non-augmented screws
Hartung (2016) [53]	4	Retrospective case series	United States	13	3.8 mo.	Periacetabular region		Impending or pathologic fracture	Cement augmented screws
Jiang (2023) [54]	2	Prospective cohort	United States	21	18.5 mo.	Periacetabular region		Impending or pathologic fracture	Cement augmented screws
Lan (2023) [55]	3	Retrospective cohort	China	11	12 mo.	All pelvic bones		Impending or pathologic fracture	Non-augmented screws
Lee (2020) [56]	2	Prospective cohort	United States	23	7 mo.	All pelvic bones	Proximal femur (4) Proximal tibia (2) Calcaneus (1)	Impending or pathologic fracture	Cement augmented screws
Lee (2023) [57]	3	Retrospective cohort	United States	56	2 mo. (median)	All pelvic bones	Sacrum (17)	Impending or pathologic fracture	Cement augmented screws
Letty (2024) [58]	3	Retrospective cohort	France	18	8 mo.	All pelvic bones		Impending or pathologic fracture	Cement augmented screws
Lozano- Calderon (2024a) [30]	3	Retrospective cohort	United States	39	6.1 mo. (median)	Periacetabular region		Impending or pathologic fracture	Photodynamic nail
Lozano- Calderon (2024b) [59]	2	Prospective cohort	United States	30	6.8 mo. (median)	Periacetabular region		Impending or pathologic fracture	Photodynamic nail
Markowitz (2025) [60]	3	Retrospective cohort	United States	15	5 mo.	All pelvic bones		Impending or pathologic fracture	Photodynamic nail
Moulin (2020) [29]	3	Retrospective cohort	France	50	–	All pelvic bones	Femoral neck (3)	Impending or pathologic fracture	Cement augmented screws
Roux (2019) [27]	3	Retrospective cohort	France	100	14.4 mo.	All pelvic bones	Sacrum	Pathologic fracture	Cement augmented screws
Wang (2024) [38]	4	Prospective case series	China	12	7.1 mo.	Periacetabular region		Impending or pathologic fracture	Both screw types
Yang (2020) [61]	3	Retrospective cohort	United States	20	7 mo.	Periacetabular region		Impending or pathologic fracture	Non-augmented screws
Yang (2022) [62]	3	Retrospective cohort	United States	22	7 mo.	All pelvic bones	Sacrum (11)	Impending or pathologic fracture	Non-augmented screws

**Table 2:** Summary of study outcomes.

First Author (Year)	Preoperative VAS Pain Score (Mean ± SD)	Postoperative VAS Pain Score (Mean ± SD)	Preoperative ECOG Score (Mean ± SD)	Postoperative ECOG Score (Mean ± SD)	Postoperative Data Collection Timepoint	Number of Complications	Number of Reoperations
Assouline (2021) [36]	1.2 ± 1.2	0.8 ± 1.7	0.7 ± 0.7	–	Unspecified	4	2
Autrusseau (2021) [28]	–	–	–	–	–	16	3
Bertholon (2022) [32]	8 ± 2.7	1.6 ± 2.5	–	–	4 weeks	2	0
Cazzato (2016) [37]	–	–	–	–	–	5	2
Cornelis (2022) [25]	3.5 ± 3.2	2.2 ± 2.3	–	–	6 months	18	0
Deschamps (2016) [26]	8.0 *	2.6 *	–	–	4 weeks	4	0
Dien (2025) [49]	–	–	–	–	–	7	1
Dussik (2023) [50]	8.0 ± 2.1	–	3.0	1.0	2 weeks	0	1
English (2021) [31]	–	–	–	–	–	3	6
Fourman (2021) [51]	6.2 ± 1.5	3.2 ± 1.5	–	–	6 weeks	2	1
Goker (2025) [52]	9.0 (median)	1.0 (median)	3.0 (median)	2.0 (median)	1 year	3	7
Hartung (2016) [53]	–	–	–	–	–	2	0
Jiang (2023) [54]	–	–	–	–	–	–	–
Lan (2023) [55]	7.9	2.8	3.5	1.8	Final, unspecified	1	1
Lee (2020) [56]	8.5 ± 1.5	2.4 ± 2.2	–	–	Unspecified	0	0
Lee (2023) [57]	–	–	3.0 ± 0.8	1.9 ± 1.1	Final, unspecified	4	4
Letty (2024) [58]	8.4 ± 1.3	2.2 ± 3.1	–	–	4 weeks	2	0
Lozano-Calderon (2024a) [30]	8.0 (median)	1.0 (median)	–	–	1 year	7	4
Lozano-Calderon (2024b) [59]	6.0 (median)	0 (median)	–	–	6 months	5	5
Markowitz (2025) [60]	9.1 ± 1.5	2.3 ± 2.8	–	–	Final, unspecified	1	2
Moulin (2020) [29]	–	–	–	–	–	5	3
Roux (2019) [27]	6.1 ± 2.5	2.1 ± 3.0	1.4 ± 0.9	1.4 ± 0.9	First, unspecified	6	2
Wang (2024) [38]	7.8 ± 1	2.3 ± 0.6	3.4 ± 0.5	1.9 ± 0.5	3 months	0	0
Yang (2020) [61]	8.8 ± 1	2.0 ± 2.2	3.4 ± 0.8	2.2 ± 0.9	3 months	1	4
Yang (2022) [62]	9.1 ± 0.9	2.8 ± 2.1	2.7 ± 0.9	2.1 ± 0.7	4 weeks	0	0

* Not included in meta-analysis because it did not report standard deviation or range.

## Data Availability

The raw data supporting the conclusions of this article will be made available by the authors on request.

## Supplementary Materials

The following supporting information can be downloaded at: https://www.mdpi.com/article/10.3390/cancers18172804/s1, The completed PRISMA 2020 checklist is provided as Supplementary File S1.

## Abbreviations

The following abbreviations are used in this manuscript:

MBD	Metastatic Bone Disease
VAS	Visual Analog Scale
ECOG	Eastern Cooperative Oncology Group
PRISMA	Preferred Reporting Items for Systematic Reviews and Meta-Analyses
MCID	Minimal Clinically Important Difference
MINORS	Methodological Index for Non-Randomized Studies
ICC	Interclass Correlation Coefficient
CI	Confidence Interval
ENT	Ear, Nose, & Throat
SD	Standard Deviation
NA	Not Available

## Appendix A

Detailed database search strategies and number of results.

**Table A1:** Database search strategies.

Database	Search Strategy	Results
MEDLINE	(((metastatic bone*) OR (exp bone metastasis) OR (metastatic cancer*) OR (metastatic lesion*) or (metastatic tumor*) OR (bone metastas*) OR (MBD)) AND ((exp pelvis) OR (pelvi*) OR (intra-pelvic) OR (acetabulum) OR (acetabular) OR (exp acetabulum) OR (peri-acetabular) OR (periacetabular)) AND ((percutaneous fixation*) OR (percutaneous reconstruct*) OR (percutaneous stabili*) OR (percutaneous treat*) OR (percutaneous guide*) OR (percutaneous surg*) OR (exp percutaneous surgery) OR (percutaneous technique*) OR (percutaneous procedure*) OR (percutaneous approach*) OR (percutaneous guide*) OR (exp minimally invasive surgery) OR (minimally invasive fixation) OR (minimally invasive reconstruct*) OR (minimally invasive stabili*) OR (minimally invasive treat*) OR (exp minimally invasive procedure) OR (minimally invasive procedure*) OR minimally invasive surg*) OR (minimally invasive technique*) OR (minimally invasive approach*) OR (minimally invasive guide*) OR (MIS) OR (cementopalst*) OR (ablation*) OR (exp cementoplasty) OR (exp tumor ablation) OR (exp ablation therapy) OR (exp radiofrequency ablation) OR (radiofrequency ablation*) OR (exp cryoablation) OR (cryoablation*)))	188
Embase	(((metastatic bone*) OR (exp bone metastasis) OR (metastatic cancer*) OR (metastatic lesion*) or (metastatic tumor*) OR (bone metastas*) OR (MBD)) AND ((exp pelvis) OR (pelvi*) OR (intra-pelvic) OR (exp acetabulum) OR (acetabulum) OR (acetabular) OR (peri-acetabular) OR (periacetabular)) AND ((percutaneous fixation*) OR (percutaneous reconstruct*) OR (percutaneous stabili*) OR (percutaneous treat*) OR (percutaneous guide*) OR (percutaneous surg*) OR (exp percutaneous surgery) OR (percutaneous technique*) OR (exp minimally invasive surgery) OR (minimally invasive reconstruct*) OR (minimally invasive stabili*) OR (minimally invasive fixation*) OR (minimally invasive treat*) OR (exp minimally invasive procedure) OR (minimally invasive surg*) OR (minimally invasive technique*) OR (minimally invasive procedure*) OR (MIS) OR (cementopalst*) OR (exp cementoplasty) OR (exp ablation therapy) OR (ablation*) OR (exp radiofrequency ablation) OR (radiofrequency ablation*) OR (exp cryoablation) OR (cryoablation*)))	495
Scopus	(((TITLE-ABS-KEY (cryoablation*)) OR (TITLE-ABS-KEY (radiofrequency AND ablation*)) OR *(TITLE-ABS-KEY (ablation*)) OR (TITLE-ABS-KEY (cementoplast*)) OR (TITLE-ABS-KEY (mis)) OR (TITLE-ABS-KEY (minimally AND invasive AND guide*)) OR (TITLE-ABS-KEY (minimally AND invasive AND approach*)) OR (TITLE-ABS-KEY (minimally AND invasive AND technique*)) OR (TITLE-ABS-KEY (minimally AND invasive AND surg*)) OR (TITLE-ABS-KEY (minimally AND invasive AND procedure*)) OR (TITLE-ABS-KEY (minimally AND invasive AND treat*)) OR (TITLE-ABS-KEY (minimally AND invasive AND stabili*)) OR (TITLE-ABS-KEY (minimally AND invasive AND reconstruct*)) OR (TITLE-ABS-KEY (minimally AND invasive AND fixation*)) OR (TITLE-ABS-KEY (percutaneous AND guide*)) OR (TITLE-ABS-KEY (percutaneous AND approach*)) OR (TITLE-ABS-KEY (percutaneous AND technique*)) OR (TITLE-ABS-KEY (percutaneous AND surg*)) OR (TITLE-ABS-KEY (percutaneous AND procedure*)) OR (TITLE-ABS-KEY (percutaneous AND treat*)) OR (TITLE-ABS-KEY (percutaneous AND stabili*)) OR (TITLE-ABS-KEY (percutaneous AND reconstruct*)) OR (TITLE-ABS-KEY (percutaneous AND fixation*))) AND ((TITLE-ABS-KEY (peri-acetabular)) OR (TITLE-ABS-KEY (periacetabular)) OR (TITLE-ABS-KEY (acetabul*)) OR (TITLE-ABS-KEY (intra-pelvic)) OR (TITLE-ABS-KEY (pelvi*))) AND ((TITLE-ABS-KEY (mbd)) OR (TITLE-ABS-KEY (bone AND metastas*)) OR (TITLE-ABS-KEY (metastatic AND tumor*)) OR (TITLE-ABS-KEY (metastatic AND lesion*)) OR (TITLE-ABS-KEY (metastatic AND cancer*)) OR (TITLE-ABS-KEY (metastatic AND bone*)))	882
Cochrane Library	(((metastatic bone*) OR (metastatic cancer*) OR (metastatic lesion*) or (metastatic tumor*) OR (bone metastas*) OR (MBD)) AND ((pelvi*) OR (intra-pelvic) OR (acetabul*) OR (periacetabular) OR (peri-acetabular)) AND ((percutaneous fixation*) OR (percutaneous reconstruct*) OR (percutaneous stabili*) OR (percutaneous treat*) OR (percutaneous procedure*) OR (percutaneous surg*) OR (percutaneous technique*) OR (percutaneous approach*) OR (percutaneous guide*) OR (minimally invasive fixation*) OR (minimally invasive reconstruct*) OR (minimally invasive stabili*) OR (minimally invasive fixation*) OR (minimally invasive treat*) OR (minimally invasive procedure*) OR (minimally invasive surg*) OR (minimally invasive technique*) OR (minimally invasive approach*) OR (minimally invasive guide*) OR (MIS) OR (cementopalst*) OR (ablation*) OR (radiofrequency ablation*) OR (cryoablation*)))	70
	Note: Asterisks are part of the database search syntax and denote truncation wildcard operators.	

## Appendix B

Detailed MINORS criteria assessment for all included studies.

**Table A2:** MINORS scores.

First Author (Year)	A Clearly Stated Aim *	Inclusion of Consecutive Patients *	Prospective Collection of Data *	Endpoint Appropriate to the Aim of the Study *	Unbiased Assessment of the Study Endpoint *	Follow-Up Period Appropriate to the Aim of the Study *	Loss to Follow-Up Less than 5% *	Prospective Calculation of the Study Size *	Total (Out of 16) *
Assouline (2021) [36]	2/2	2/2	0/0	2/2	1/1	2/2	1/1	0/0	10/10
Autrusseau (2021) [28]	2/2	2/2	0/0	2/2	1/1	2/1	1/1	0/0	10/9
Bertholon (2022) [32]	2/2	2/2	2/2	2/2	1/1	1/1	2/2	0/0	12/12
Cazzato (2016) [37]	2/2	2/2	0/0	2/2	1/1	2/2	1/1	0/0	10/10
Cornelis (2022) [25]	2/2	2/2	0/0	2/2	1/1	2/2	0/0	0/0	9/9
Deschamps (2016) [26]	2/2	2/2	0/0	2/2	1/1	2/2	0/0	0/0	9/9
Dien (2025) [49]	2/2	2/2	0/0	2/2	1/1	2/2	2/2	0/0	11/11
Dussik (2023) [50]	2/2	1/2	0/0	2/2	1/1	2/2	2/2	0/0	10/11
English (2021) [31]	2/2	2/2	0/0	2/2	1/1	1/2	2/2	0/0	10/11
Fourman (2021) [51]	0/0	2/2	0/0	2/2	1/1	1/1	1/2	0/0	7/8
Goker (2025) [52]	2/2	2/2	0/0	2/2	1/1	2/2	2/2	0/0	11/11
Hartung (2016) [53]	2/2	2/2	0/0	2/2	1/1	2/2	1/1	0/0	10/10
Jiang (2023) [54]	2/2	0/1	2/2	2/2	2/1	2/2	1/1	0/0	11/11
Lan (2023) [55]	2/2	1/2	0/0	2/2	1/1	2/2	2/2	0/0	11/11
Lee (2020) [56]	2/2	2/2	2/2	2/2	1/1	2/2	2/2	0/0	13/13
Lee (2023) [57]	2/2	2/2	0/0	2/2	1/1	1/1	1/1	0/0	9/9
Letty (2024) [58]	2/2	2/2	0/0	2/2	1/1	2/2	2/2	0/0	11/11
Lozano-Calderon (2024a) [30]	2/2	2/2	2/2	2/2	1/1	2/1	0/0	0/0	11/10
Lozano-Calderon (2024b) [59]	2/2	2/2	0/0	2/2	1/1	2/1	0/0	0/0	9/8
Markowitz (2025) [60]	2/2	2/2	0/0	2/2	1/1	2/2	2/2	0/0	11/11
Moulin (2020) [29]	2/2	2/2	0/0	2/2	1/1	2/2	2/2	0/0	11/11
Roux (2019) [27]	2/2	2/2	0/0	2/2	1/1	1/2	2/1	0/0	10/10
Wang (2024) [38]	2/2	2/2	2/2	2/2	1/1	2/1	2/2	0/0	13/12
Yang (2020) [61]	2/2	2/2	0/0	2/2	1/1	1/2	2/2	0/0	8/11
Yang (2022) [62]	2/2	2/2	0/0	2/2	1/1	2/1	2/2	0/0	9/10

* Values reported as score assigned by reviewer one/score assigned by reviewer two.
